# Restaurant occupational exposure affects the profiles of oral and gut pathobiomes and resistomes

**DOI:** 10.3389/fmicb.2026.1771459

**Published:** 2026-02-16

**Authors:** Mengyu Wang, Xueqin Li, Xiao Liu, Ying Ye, Peng Zhou, Yifan Liu, Liudan Zhu, Wei Wei, Zhenpeng Li, Zhe Li, Ruiheng Wu, Yao Peng, Ziyu Liu, Xin Lu, Jiayong Zhao, Biao Kan

**Affiliations:** 1 School of Public Health, Cheeloo College of Medicine, Shandong University, Jinan, Shandong, China; 2 National Key Laboratory of Intelligent Tracking and Forecasting for Infectious Diseases, National Institute for Communicable Disease Control and Prevention, Chinese Center for Disease Control and Prevention, Beijing, China; 3 Institute of Infectious Disease Prevention and Control, Henan Center for Disease Control and Prevention, Zhengzhou, China; 4 Zhengzhou Center for Disease Control and Prevention, Zhengzhou, China

**Keywords:** gut, oral, pathogens, plasmids, Rank_I ARGs, restaurant occupational exposures, *Salmonella*

## Abstract

**Introduction:**

Restaurant occupational exposure refers to contact with food-processing environments, raw materials, and customers, which may influence the composition of the human microbiome. Differences and associations between human oral and gut pathobiome and their resistomes under restaurant occupational exposure remain unclear. We conducted a comprehensive metagenomic analysis of paired oral and fecal samples from Front-of-House (FOH) workers and Back-of-House (BOH) workers to elucidate the effects of occupational exposure in the restaurant environment on oral and gut pathobiome, antimicrobial resistance genes (ARGs), virulence factors (VFs), and mobile genetic elements (MGEs).

**Methods:**

We collected the oral and fecal samples from 35 FOH and 37 BOH workers across 24 Chinese restaurants in Zhengzhou, Henan, China. The diversity and relative abundances of microbial species, ARGs, VFs, and MGEs were compared. Clonal strains from paired oral and fecal samples were analyzed. The serovars of *Salmonella* were determined using the ucgMLST. Finally, we used the O2PLS method to explore relationships among ARG subtypes, bacterial communities (species-level), MGEs (subtype-level), and plasmids.

**Results:**

The gut microbiome acts as the primary reservoir, exhibiting greater alpha diversity and a higher burden of pathogens/resistomes (including high-risk Rank_I genes). In contrast, the oral microbiome was more sensitive to occupational differences. Significant beta diversity variations in microbiomes, antimicrobial resistance genes (ARGs), and virulence factors were observed exclusively in oral samples. Notably, *Salmonella* Typhimurium was significantly more prevalent in the oral cavity of BOH workers (*R*^2^ = 0.032, *p* = 0.047), indicating their potential role as intermediaries in foodborne pathogen transmission. Strain-level analysis confirmed that clonal strains of the opportunistic pathogen and probiotics were shared between the oral cavity and the gut. O2PLS analysis identified plasmids as the main correlates of ARGs.

**Discussion:**

While the gut serves as the primary reservoir for pathogens/resistomes, restaurant occupational exposure distinctly shapes oral microbial/resistome profiles, underscoring the critical need for reinforced hygiene management, particularly for BOH workers, to mitigate pathogen and resistance transmission.

## Introduction

The human microbiome is a complex and dynamic ecosystem that varies across body sites and plays critical roles in maintaining health and mediating interactions with the environment (Cho and Blaser, 2012). Among these, the oral and gut microbiomes constitute the most complex and densely populated microbial communities in the human body, with dysbiosis linked to a wide range of systemic diseases (Tan et al., 2023). Recent evidence highlights the complex interactions between the oral and gut microbiota (Xu et al., 2025; Maki et al., 2021). The gut microbiome is characterized by high species richness and a dense network of microbial interactions that collectively contribute to nutrient metabolism, immune modulation, and maintenance of systemic homeostasis (Lozupone et al., 2012). In contrast, the oral microbiome is highly dynamic and directly exposed to dietary and environmental influences (Nath et al., 2025). Despite being physiologically connected, they differ markedly in microbial diversity, ecological stability, and responsiveness to external influences (Kitamoto et al., 2020). Understanding their relationships is therefore critical for elucidating how external factors influence human microbial homeostasis.

Occupational exposure represents an important but often overlooked determinant of microbiome composition (Van Gompel et al., 2020). Previous studies have demonstrated that occupational exposure is linked to the occurrence of specific antimicrobial-resistant bacteria (Van Gompel et al., 2020; Sun et al., 2020). Moreover, occupational exposure serves as a critical pathway through which socioeconomic and gendered power relations shape differential risks of resistant infections (Davis et al., 2025). Individuals in specific professions from livestock farmers to healthcare workers face heightened exposure to antimicrobials and resistant pathogens, often as a direct consequence of their livelihood practices and working conditions (Atterby et al., 2019; Pearson and Chandler, 2019). Although these insights underscore the role of occupational exposure in shaping human microbiome and antimicrobial resistance (AMR), the extent to which restaurant occupational environments influence oral and gut microbiota remains poorly understood. In restaurant settings, two major occupational groups can be distinguished: Back-of-House (BOH) workers (e.g., chefs, cooks, dishwashers) and Front-of-House (FOH) workers (e.g., wait-staff, cashiers). BOH workers are routinely exposed to pathogens through direct contact with raw food materials (including eggs, meat, seafood, and vegetables), food-processing environments, and contaminated cookware surfaces. Such exposure may introduce exogenous bacteria, including opportunistic pathogens and antibiotic-resistant strains into the human microbiome (Ho et al., 2014; Zegeye et al., 2025). In contrast, frequent customer interactions, serving food, and clearing used tableware expose FOH workers to human-sourced bacteria via aerosol/droplets and fomite-to-hand transmission. These differences in exposure sources and transmission routes likely contribute to the observed variations in the pathobiome and resistome between the two occupational groups. In addition, the presence of antibiotic residues in the restaurant environments to which BOH workers are routinely exposed imposes selective pressure that enhances the activity of mobile genetic elements (MGEs), thereby facilitating horizontal gene transfer and promoting the persistence of AMR determinants (Aworh et al., 2021; Sun et al., 2017). At the same time, MGEs and virulence factors (VFs) contribute to the complexity of microbial dynamics (Wang et al., 2022; Bottery, 2022). Consequently, BOH workers may serve as biological bridges for microbial and AMR dissemination between the food chain, occupational settings, and the general population.

In this study, we conducted a comprehensive metagenomic analysis of paired oral and fecal samples collected from FOH and BOH workers to investigate the differential impacts of occupational exposure on microbial diversity, resistome profiles, MGEs, and VFs. By integrating analyses across the oral and gut microbiomes, this study aimed to establish a foundational understanding of the effects of restaurant occupational exposure on the pathobiome and resistome profiles of FOH and BOH workers.

## Materials and methods

### Sample collection

Our study involved 24 Chinese restaurants in Zhengzhou, Henan, China, during August 2024. These restaurants reflected the common kitchen profiles of Chinese restaurant, featuring omnivore-oriented menus that included various meat and vegetable dishes. Participants were classified into two occupational groups: Front-of-House (FOH) workers (including wait-staff, cashiers, and other service personnel with frequent customer contact) and Back-of-House (BOH) workers (including chefs, cooks, dishwashers, and food preparation staff working primarily in kitchen areas with routine exposure to raw food materials such as meat, seafood, eggs, and vegetables, along with food-processing environments and contaminated cookware surfaces) (Smith et al., 2012). Inclusion criteria were: (1) age between 18 and 65 years; (2) provision of paired oral and fecal samples. Exclusion criteria included diarrhea or antibiotic use within 7 days prior to sampling. The final cohort comprised 35 FOH workers and 37 BOH workers, yielding 144 samples (72 paired oral–fecal sets). This study was conducted in accordance with the Declaration of Helsinki and was approved by the Ethics Committee of the National Institute for Communicable Disease Control and Prevention, Chinese Center for Disease Control and Prevention (Approval number: ICDC-2022001). Written informed consent was obtained from all participants.

Oral (buccal mucosa swabs, T) and fecal (S) samples were collected by trained field staff from the Zhengzhou Center for Disease Control and Prevention. Oral samples were obtained by rubbing the inside of both cheeks with two swabs, while fecal samples were self-collected by participants using sterile, screw-cap fecal containers provided in advance (Flemer et al., 2018). Fecal samples were collected within 24 h of oral sampling to ensure temporal proximity. All samples were temporarily stored at 4 °C on-site and transported to the laboratory in 24 h. DNA extraction was performed immediately after the sample arrived at the laboratory.

### Metagenomic sequencing

Genomic DNA was extracted from fecal samples and oral swabs using the QIAamp PowerFecal DNA Kit (Qiagen, Germany) and QIAamp DNA Mini Kit (Qiagen, Germany), respectively, following the manufacturers’ protocols. The DNA concentrations were quantified using a Qubit 4™ fluorometer with the Qubit dsDNA HS Assay kit (Thermo Fisher Scientific, MA, United States). Libraries were constructed using the MGIEasy FS DNA Library Prep Set (MGI, China). A paired-end library with an insert size of ~350 bp was constructed for each sample and sequenced on the MGISEQ-2000RS platform (MGI) (Lu et al., 2024). The metagenomic sequencing of the 144 samples generated a total of 962.783 Gb (6.7 ± 0.6Gb per sample after quality control).

### Microbiome and genomic profiling

The raw reads were processed using fastp, which filtered low-quality bases and kept only reads with >50 bp in size. To remove human DNA contamination, the sequencing data were mapped to the human genome (hg38) using Bowtie2 (v2.3.5.1). Taxonomic profiling of the metagenomic sequencing samples was performed using MetaPhlAn v4.0.2 with default parameters. Clonal strains from paired oral and fecal samples were analyzed using StrainPhlAn v4.0.2, following the instructions.^1^ The strain-level heterogeneity of *Salmonella* isolates in samples were analyzed using ucgMLST.^2^ Reference genomes representing various *Salmonella* serovars were incorporated for comparative analysis to determine the serovar of *Salmonella* identified in this study. ARGs were annotated using the ARGs-OAP v2.0 pipeline against the SARG 2.0 database, applying a sequence identity threshold of 80%, an e-value cutoff of 1e-10, and a coverage threshold of 70% (Yang et al., 2016). Additionally, VFs and MGEs were identified by the Virulence Factor Database (VFDB) and the Mobile Genetic Element Database, respectively, using the same ARGs-OAP v2.0 framework with identical parameters (80% identity and 70% coverage). The relative abundances of ARGs, VFs, and MGEs were expressed as copies per prokaryotic cell, calculated by normalizing the target gene copy numbers to the total 16S rRNA gene copies. The risk rank of ARGs were evaluated using arg_ranker (v3.0.1) (Zhao et al., 2025).

### Statistical analysis

Differential analyses were conducted using Wilcoxon rank-sum tests or Kruskal–Wallis tests. Fisher’s exact test was employed when more than 20% of the cells in the contingency table had an expected count of less than five. For comparisons involving more than two groups, multiple testing was performed with the R package FSA (v0.9.3), with *p*-values adjusted via the Bonferroni correction method to account for multiple testing. Alpha and beta diversity of species were calculated using the “adonis” function from the R package vegan (v2.6.4) and the “pairwise. Adonis” function from the R package pairwiseAdonis (v0.4.1), respectively (Feng et al., 2025). Differences in relative abundance of the microbial, ARGs, and VFs features between FOH and BOH workers were determined by linear discriminant analysis effect size (LDA > 2) (LEfSe) (Segata et al., 2011). We further applied the O2PLS method to explore the relationships between ARGs (subtype-level) and bacterial communities (BACs, species-level), MGEs (subtype-level), and plasmids, aiming to characterize the main components underlying these associations. Variation within the datasets (ARGs-BACs, ARGs-MGEs, ARGs-Plasmids) was decomposed by O2PLS in the R package OmicsPLS (v2.02) (el Bouhaddani et al., 2018).

## Results

### Distinctive features of oral and gut microbiota and their associations with restaurant occupational exposure

**Figure 1:**
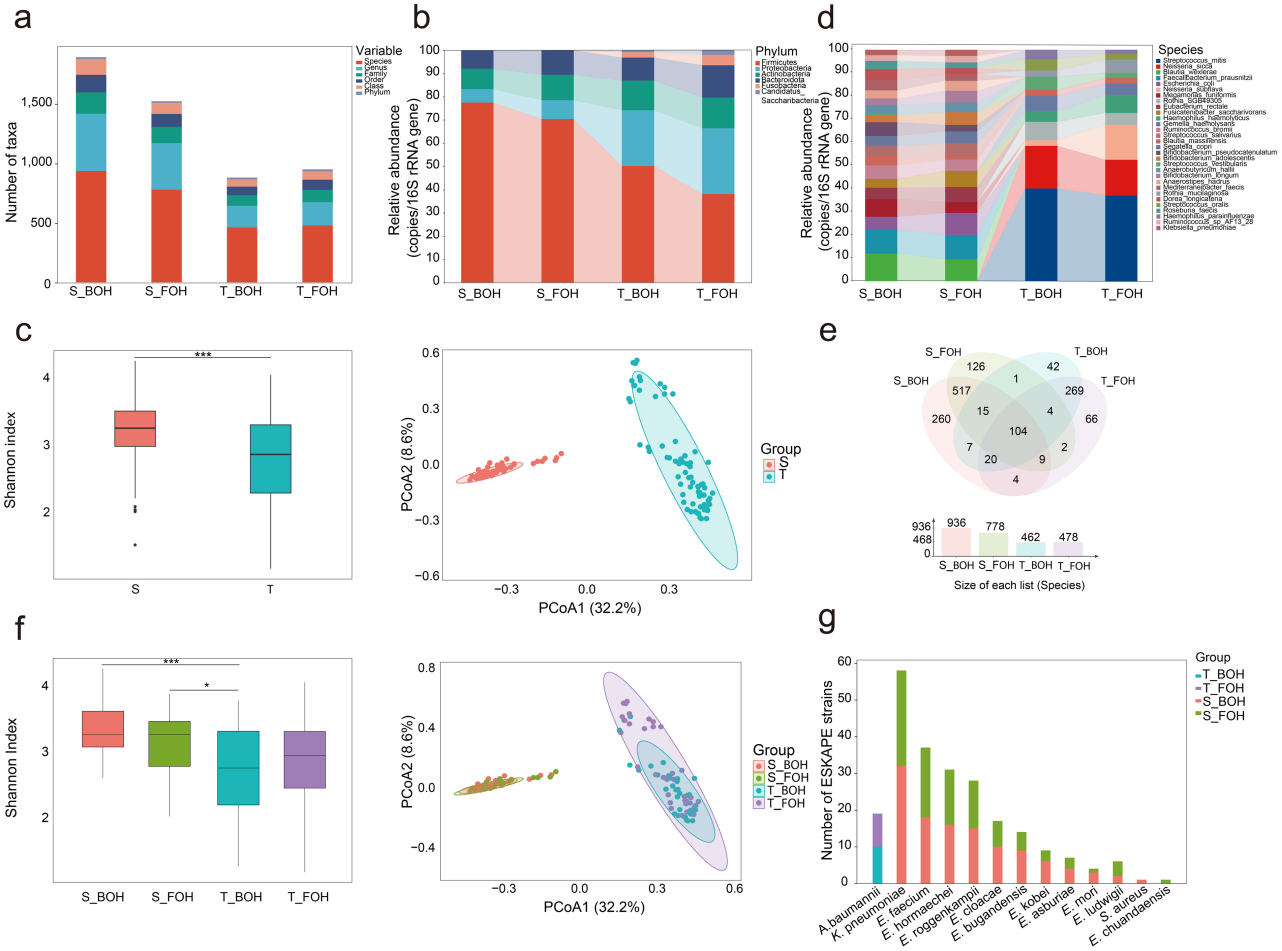
The microbiome profiles of the 144 samples (S: fecal, T: oral, S_BOH: fecal samples collected from back-of-house workers, S_FOH: fecal samples collected from front-of-house workers, T_BOH: oral samples collected from back-of-house workers, T_FOH: oral samples collected from front-of-house workers) (*0.01 < *p* < 0.05, ***p < 0.001). **(a)** Microbial profiles of the four groups at the phylum, class, order, family, genus, and species levels. **(b)** Microbial profiles at the phylum level for the four groups (with a total abundance ≥1% in all four groups). **(c)** The Shannon indices of species (*χ*^2^ = 13.63, *p* = 0.0002) and principal coordinate analysis (PCoA) of the species (*R*^2^ = 0.31, *p* = 0.001) in the oral and fecal samples. **(d)** Microbial profiles at the species level for the four groups (with a total abundance ≥3% across all samples). **(e)** Venn diagram of species level overlapped among the four groups. **(f)** The Shannon indices of species (S_BOH&S_FOH: *Z* = 1.04, *p_adj_* > 0.05; S_BOH&T_BOH: *Z* = 3.82, *p_adj_* = 0.0008; S_BOH&T_FOH: *Z* = 2.43, *p_adj_* = 0.09; S_FOH&T_BOH: *Z* = 2.72, *p_adj_* = 0.039; S_FOH&T_FOH: *Z* = 1.37, *p_adj_* > 0.05; T_BOH&T_FOH: *Z* = −1.33, *p_adj_* > 0.05) and PCoA of the species (S_BOH&S_FOH: *R*^2^ = 0.01, *p_adj_* = 0.461; S_BOH&T_BOH: *R*^2^ = 0.34, *p_adj_* = 0.0015; S_BOH&T_FOH: *R*^2^ = 0.31, *p_adj_* = 0.0015; S_FOH&T_BOH: *R*^2^ = 0.32, *p_adj_* = 0.0015; S_FOH&T_FOH: *R*^2^ = 0.30, *p_adj_* = 0.0015; T_BOH&T_FOH: *R*^2^ = 0.03, *p_adj_* = 0.0144) in the four groups. **(g)** The distribution of the ESKAPE pathogens across the four groups.

Among 72 paired oral and fecal samples, the oral microbiome comprised 19 phyla, 76 classes, 91 orders, 110 families, 220 genera, and 544 species, while the fecal microbiome consisted of 15 phyla, 147 classes, 164 orders, 199 families, 550 genera, and 1,069 species (Figure 1a). At the phylum level, Firmicutes, Proteobacteria, Actinobacteria, Bacteroidetes, and Fusobacteria accounted for 97.36% of the total abundance in oral samples, while Firmicutes, Proteobacteria, Actinobacteria, and Bacteroidetes together accounted for 99.75% in fecal samples, with both exceeding 95% of the total abundance (Figure 1b). The fecal samples exhibited significantly higher species alpha diversity than the oral samples (*χ*^2^ = 13.63, *p* = 0.0002) (Figure 1c). Significant differences were also observed in beta diversity (*R*^2^ = 0.31, *p* = 0.001) (Figures 1c,d). At the population level, the oral and fecal samples overlapped 166 species-level (Figure 1e). Among these, 129 species-level and 141 strain-level taxa were observed in at least one paired oral-fecal sample.

From the 141 strain-level taxa, we selected taxa with a total abundance greater than 100 and suitable for phylogenetic tree construction for strain-level analysis using StrainPhlAn (Supplementary Figure 1). Ultimately, strain-level analysis revealed that clonal strains were identified in four oral-fecal pairs for s_*Streptococcus salivarius*|t_SGB8007_group, two pairs for *s_Haemophilus parainfluenzae*|t_SGB9712, and one pair for *s_Veillonella parvula*|t_SGB6939 (SNP < 5) (Supplementary Figures 1g–i). The sharing/transfer of these strains between oral and gut indicated that the oral and intestinal microbiota are not independent. For both the oral and fecal samples, no statistically significant differences in species alpha diversity were detected between the FOH and BOH workers (all *p_adj_* > 0.05); however, species beta diversity differed significantly between the two occupations only in the oral samples (*R*^2^ = 0.03, *p* = 0.0144) (Figure 1f). Subsequently, we performed LEfSe analysis on species-level taxa from the oral samples of the FOH and BOH workers to investigate the clustering patterns of bacterial strains between the two occupational groups. Distinct microbial biomarkers were observed between groups. Taxa enriched in the BOH workers included *Streptococcus vestibularis* (LDA scores = 4.06), *Rothia SGB49305* (LDA scores = 3.99), *Streptococcus gordonii* (LDA scores = 3.52), and *Streptococcus oralis* (LDA scores = 3.98). Conversely, *Neisseria subflava* (LDA scores = 4.52), *Porphyromonas bobii* (LDA scores = 3.84), and *Fusobacterium periodonticum* (LDA scores = 3.75) were significantly associated with the FOH workers (Supplementary Figure 2). Notably, among the species detected in both oral and fecal samples, several were linked to animal-derived foods exposure. For instance, *Weissella viridescens*, an opportunistic pathogen capable of causing potentially fatal infections (Jamwal et al., 2024) and *Lactococcus carnosus* associated with fermented meat (Hilgarth et al., 2020; Liu et al., 2025; Liu et al., 2021; Fusco et al., 2023)were exclusively detected in the BOH workers (S_BOH and T_BOH) but were not detected in FOH workers (S_FOH and T_FOH).

### Profiles of pathogens and VFs in oral and gut microbiomes under restaurant occupational exposure

We further investigated the distribution of clinically relevant pathogens, and identified several species corresponding to the ESKAPE (*Enterococcus faecium*, *Staphylococcus aureus*, *Klebsiella pneumoniae*, *Acinetobacter baumannii*, *Pseudomonas aeruginosa*, and *Enterobacter* species) group. Notably, although ESKAPE pathogens were identified in both oral and fecal samples, their prevalence and diversity were significantly higher in the fecal (13 species) than in the oral cavity (one species) (Figure 1g). This suggests that the gut was more important for harboring ESKAPE pathogens. No statistically significant difference was observed in the carriage rate of ESKAPE pathogens between the two occupational groups, with rates of 91.9% (34/37) in the BOH workers and 80.0% (28/35) in the FOH workers (*χ*^2^ = 2.13, *p* = 0.19).

**Figure 2:**
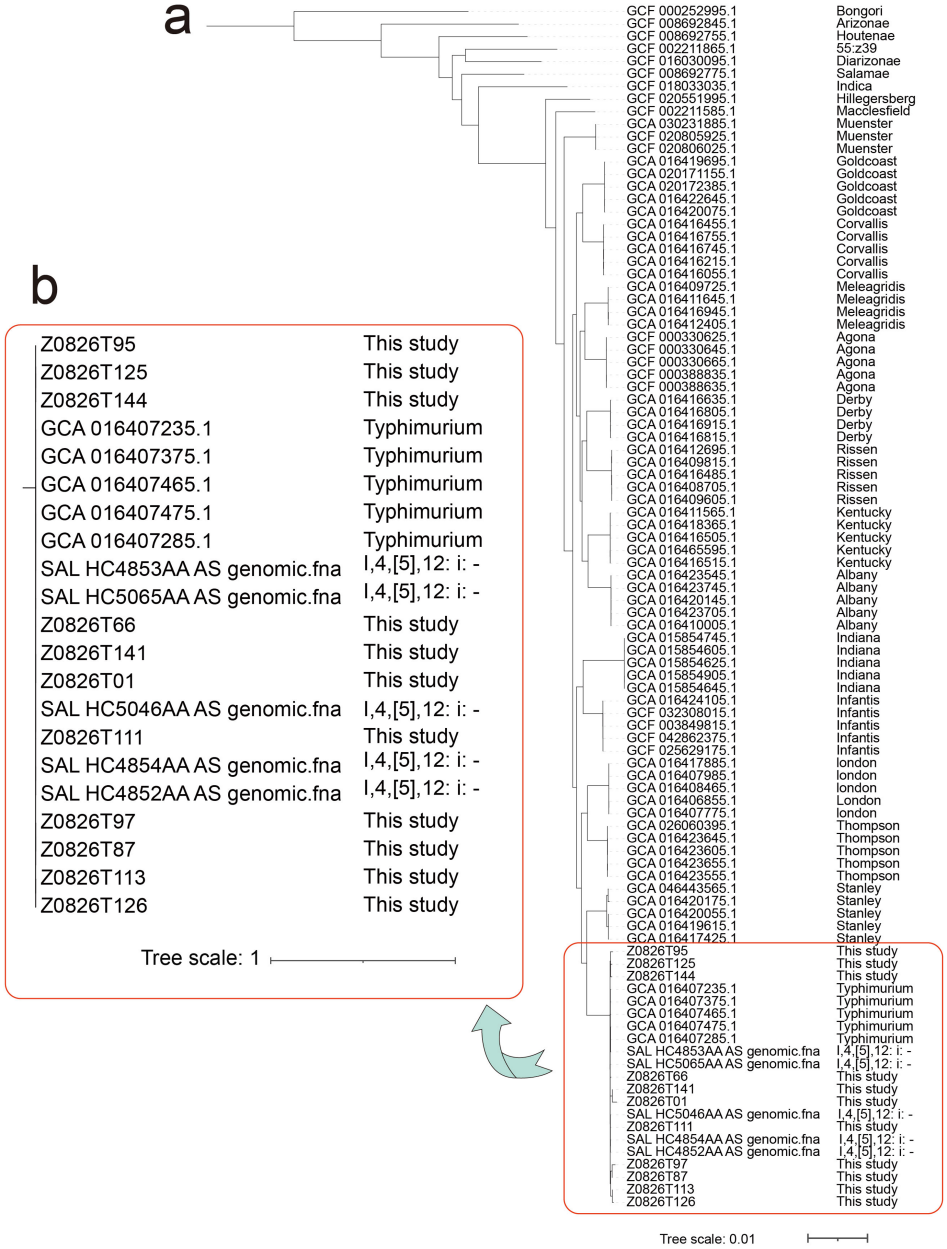
Phylogenetic analysis of *Salmonella*-positive samples based on ucgMLST, including sequences from this study, reference genomes from the RefSeq database, and the genomes of representative *Salmonella* serovars obtained from NCBI (S: fecal, T: oral). **(a)** Phylogenetic analysis of multiple *Salmonella* serovars together with the *Salmonella*-positive samples from this study. **(b)** Fine phylogenetic analysis of *Salmonella* Typhimurium and *Salmonella* 1,4,[5],12:i:- serovars together with the *Salmonella*-positive samples from this study.

*Salmonella*, a major foodborne pathogen, imposes a substantial global disease burden in both developing and developed countries (Majowicz et al., 2010). In this study, *Salmonella enterica* (LDA scores = 2.24) was observed to be enriched in the BOH workers. We subsequently performed a comprehensive analysis of *Salmonella enterica* identified within the samples. The results indicated *Salmonella enterica* was identified in a total of 11 samples (nine from the BOH workers and two from the FOH workers, *R*^2^ = 0.032, *p* = 0.047) and exhibited abundance only in the oral samples, with no detection in fecal samples. To elucidate the serovar and phylogenetic relationships of these isolates, a ucgMLST-based approach was employed for all *Salmonella enterica* obtained in this study. This analysis incorporated multiple reference genomes of various known *Salmonella* serovars from the NCBI database to provide a robust comparative framework (Figure 2a). Phylogenetic reconstruction suggested that the detected *Salmonella* strains in this study are likely *Salmonella* Typhimurium (*S.* Typhimurium or 1,4,[5],12:i:-) (Figure 2b). *S.* Typhimurium and 1,4,[5],12:i:- are among the most frequently reported *Salmonella* serovars worldwide and have been commonly isolated from raw animal-derived foods, particularly poultry and pork, in both global surveillance and national food safety monitoring studies in China (Yin et al., 2022; Yang et al., 2023; Li et al., 2025; Campos et al., 2016).

Beyond the presence of specific pathogens, VF profiles was analyzed. A total of 1,201 VFs were identified. Likewise, fecal samples exhibited significantly higher alpha diversity than oral samples (*χ*^2^ = 74.621, *p* < 0.001). Beta diversity analysis also revealed significant differences between the two sample types (*R*^2^ = 0.32, *p* = 0.001) (Supplementary Figure 3a), with *ibeC* dominating in fecal samples and *psaA* in oral samples (Supplementary Figure 3b). A total of 913 VFs were overlapped between oral and fecal samples. Notably, 288 VFs were exclusively detected in oral samples, whereas no VFs were found only in fecal samples, suggesting that restaurant occupational exposure may predominantly influence the VFs of oral microbiota. Given the observed sample-specific patterns, we next examined whether occupational groups differed in their VF composition. The total VFs abundance and VFs alpha did not differ significantly between occupational groups (all *p_adj_* > 0.05), whereas the beta diversity showed a significant difference between two occupational groups for the oral samples (Supplementary Figures 3c,d). Distinct VFs biomarkers were observed between groups. Taxa enriched in the BOH workers included *iga* (LDA scores = 3.58). Conversely, *lipA* (LDA scores = 4.15) and *lipB* (LDA scores = 4.12) were significantly associated with the FOH workers (Supplementary Figure 4).

### Oral and gut resistome profiles and MGEs and their association with restaurant occupational exposure

**Figure 3:**
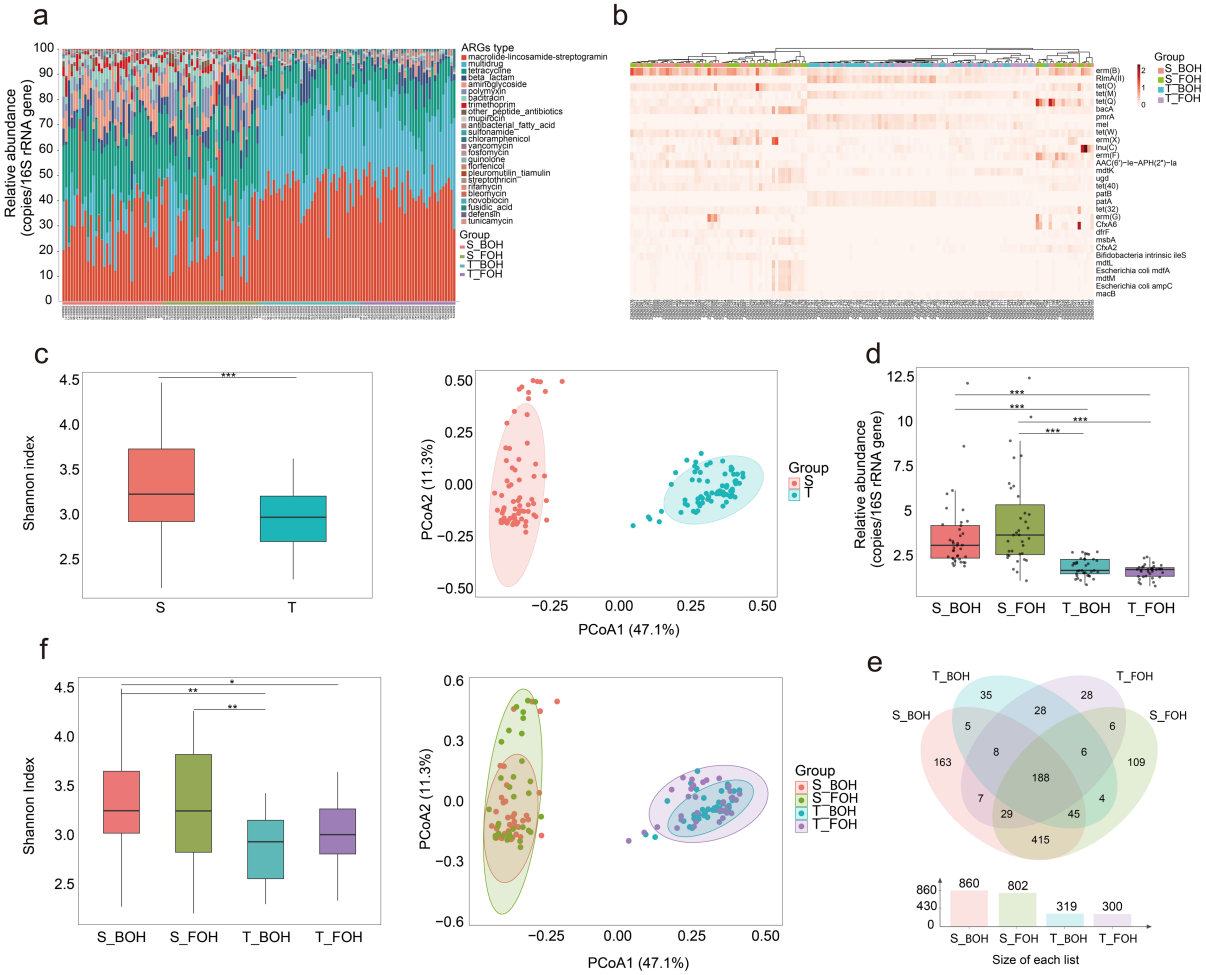
The resistome profiles of the 144 samples (S: fecal, T: oral, S_BOH: fecal samples collected from Back-of-House workers, S_FOH: fecal samples collected from Front-of-House workers, T_BOH: oral samples collected from Back-of-House workers, T_FOH: oral samples collected from Front-of-House workers) (*0.01 < *p* < 0.05, **p < 0.01, ***p < 0.001). **(a)** The proportion of antimicrobial resistance genes (ARGs) types among four groups. **(b)** The relative abundance of the top 30 ARG subtypes across all samples. **(c)** The Shannon indices of ARGs (*χ*^2^ = 15.96, *p* < 0.001) and principal coordinate analysis (PCoA) of the ARGs (*R*^2^ = 0.45, *p* = 0.001) in the oral and fecal samples. **(d)** The ARGs abundance in the four groups. The abundance of ARGs was calculated as a “copy of ARG per copy of 16S rRNA gene” (S_BOH&S_FOH: *Z* = −0.55, *p_adj_* = 0.58; S_BOH&T_BOH: *Z* = −5.47, *p_adj_* < 0.001; S_BOH&T_FOH: *Z* = −6.61, *p_adj_* < 0.001; S_FOH&T_BOH: *Z* = −5.94, *p_adj_* < 0.001; S_FOH&T_FOH: *Z* = −7.06, *p_adj_* < 0.001; T_BOH&T_FOH: *Z* = −1.23, *p_adj_* = 0.44). **(e)** Venn diagram of ARG subtypes overlapped among the four groups. **(f)** The Shannon indices of ARG subtypes (S_BOH&S_FOH: *Z* = 0.59, *p_adj_* > 0.05; S_BOH&T_BOH: *Z* = 3.76, *p_adj_* = 0.0005; S_BOH&T_FOH: *Z* = 2.48, *p_adj_* = 0.0398; S_FOH&T_BOH: *Z* = 3.12, *p_adj_* = 0.0054; S_FOH&T_FOH: *Z* = 1.86, *p_adj_* = 0.1888; T_BOH&T_FOH: *Z* = −1.24, *p_adj_* = 0.6497) and PCoA of the ARG subtypes (S_BOH&S_FOH: *R*^2^ = 0.02, *p_adj_* = 0.263; S_BOH&T_BOH: *R*^2^ = 0.48, *p_adj_* = 0.001; S_BOH&T_FOH: *R*^2^ = 0.48, *p_adj_* = 0.001; S_FOH&T_BOH: *R*^2^ = 0.45, *p_adj_* = 0.001; S_FOH&T_FOH: *R*^2^ = 0.44, *p_adj_* = 0.001; T_BOH&T_FOH: *R*^2^ = 0.03, *p_adj_* = 0.047) in the four groups.

A total of 22 and 25 types of ARGs, encompassing 389 and 982 subtypes, were identified in oral and fecal samples, respectively. In both oral and fecal samples, the top four most abundant ARG types are macrolide-lincosamide-streptogramin (MLS) [*erm(B)*, *RlmA(II)*, etc.], multidrug (*CfxA6*, *CfxA2*, etc.), tetracycline [*tet(O)*, *tet(M)*, etc.], and beta-lactam [*tet(O)*, *tet(M)*, *tet(Q)*, etc.] (Figures 3a,b). Oral and fecal samples exhibited distinct resistome profiles. Fecal samples harbored a significantly higher abundance of ARGs than oral samples (*χ*^2^ = 15.96, *p* < 0.001), indicating a greater resistome burden in the gut compared to the oral environment. In addition, beta diversity analysis revealed distinct clustering patterns between oral and fecal samples (*R*^2^ = 0.45, *p* = 0.001; Figure 3c). This finding was further supported by significant differences in ARGs abundance profiles (Figure 3d). At the population level, a total of 22 ARG types (298 subtypes) overlapped between oral and fecal samples, while 91 ARG subtypes (13 types) were exclusively identified in oral samples and another 687 distinct ARG subtypes (18 types) were found only in fecal samples (Figure 3e). The tetracycline resistance genes *tet(X4)* and *tet(X5)*, which confer resistance to last-resort antibiotics such as tigecycline, were detected exclusively in fecal samples but were absent in oral samples. In the FOH and BOH workers, no statistically significant differences in alpha diversity of ARGs were found (all *p_adj_* > 0.05); however, significant differences in beta diversity were detected between the two occupational groups within the oral samples (*R*^2^ = 0.032, *p* = 0.047) (Figure 3f). Distinct ARGs biomarkers were observed between groups. Taxa enriched in the BOH workers included macrolide-lincosamide-streptogramin*-RlmA(II)* (LDA scores = 4.09) and multidrug*-pmrA* (LDA scores = 4.06). Conversely, macrolide-lincosamide-streptogramin*-macB* (LDA scores = 3.58) and macrolide-lincosamide-streptogramin*-macA* (LDA scores = 3.39) were significantly associated with the FOH workers (Supplementary Figure 5). Notably, five ARGs (*amrA*, *abcA*, *cmx*, *qacB*, and *mcr-1.5*) were found exclusively in 18 samples from the S_BOH and T_BOH workers, whereas six ARGs [*bla*_TEM-121_, *bla*_TEM-55_, *ere(A)*, *vanB*, *bla*_TEM-149_, *bla*_TEM-199_] were found exclusively in 10 samples from the S_FOH and T_FOH workers, suggesting distinct transmission routes for the different ARG flow.

**Figure 4:**
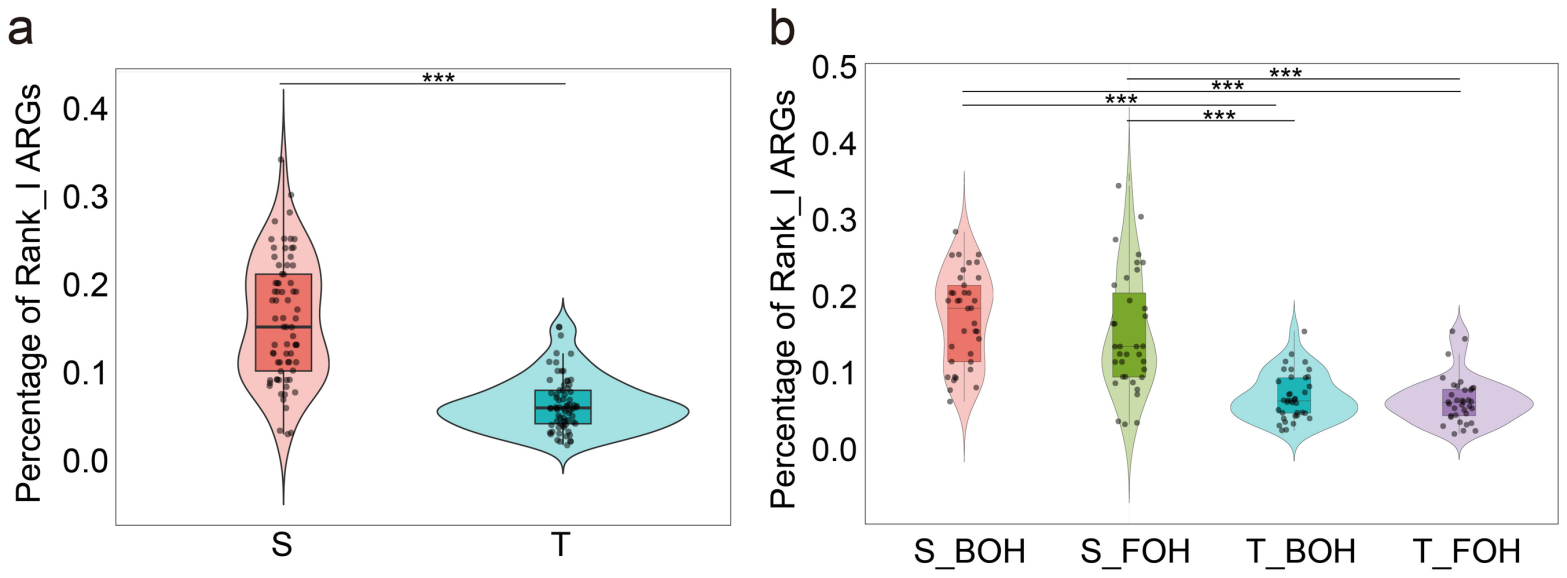
The percentages of Rank_I antimicrobial resistance genes (ARGs) among the 144 samples (S: fecal, T: oral, S_BOH: fecal samples collected from back-of-house workers, S_FOH: fecal samples collected from front-of-house workers, T_BOH: oral samples collected from back-of-house workers, T_FOH: oral samples collected from front-of-house workers). **(a)** The percentages of Rank_I ARGs among the oral and fecal samples (*w* = 4,675, *p* < 0.001). **(b)** The percentages of Rank_I ARGs in the four groups (S_BOH&S_FOH: *Z* = 1.28, *p_adj_* = 0.241; S_BOH&T_BOH: *Z* = 6.31, *p_adj_* < 0.001; S_BOH&T_FOH: *Z* = 6.81, *p_adj_* < 0.001; S_FOH&T_BOH: *Z* = 4.94, *p_adj_* < 0.001; S_FOH&T_FOH: *Z* = 5.45, *p_adj_* < 0.001; T_BOH&T_FOH: *Z* = 0.58, *p_adj_* = 0.559).

In order to assess the risk level of ARGs, they are divided into four grades (Rank I, Rank II, Rank III, and Rank IV) according to human-associated enrichment, gene transferability, and host pathogenicity. All 144 samples were harbored 56 ARGs of Rank I, such as *erm*(B), *tet*(M), which are characterized by high mobility, broad niche adaptability, and significant health risks (Zhao et al., 2025). The proportion of Rank_I ARGs (Rank_I_per) in fecal samples was significantly higher than that in oral samples (*w* = 4,675, *p* < 0.001) (Figure 4a). A total of 30 Rank_I genes were common to both oral and fecal samples. In contrast, only one Rank_I ARG [*tet(G)*] was exclusively identified in the oral group, whereas the fecal group exhibited a substantially greater number of unique Rank_I ARGs (n = 23), including *bla*_CTX-M-55_, a plasmid-mediated gene known for its high transferability across bacterial strains and hosts (Misumi et al., 2023). In contrast, no statistically significant difference was observed in Rank_I_per ARG between the FOH and BOH groups (all *p_adj_* > 0.05) (Figure 4b). We identified the core high-risk resistome common to all groups. Despite the different occupational and sampling locations, a set of 23 Rank_I ARGs was universally detected across all four groups (S_FOH, S_BOH, T_FOH, and T_BOH), highlighting a baseline public health risk attributable to a stable and widely disseminated reservoir of high-mobility ARGs. Furthermore, proportion analysis of ARGs across different ranks (Rank_II and Rank_III) also revealed that these ARGs were significantly higher in fecal samples than in oral samples (all *p_adj_* < 0.05) (Supplementary Figures 6a,b), whereas the proportion of Rank_IV ARGs was higher in oral samples than in fecal samples (*w* = 66, *p* < 0.001) (Supplementary Figure 6c). However, for all four ARG Rank grades, no statistically significant differences were observed between the FOH and BOH groups (all *p_adj_* > 0.05) (Supplementary Figures 6d–f).

**Figure 5:**
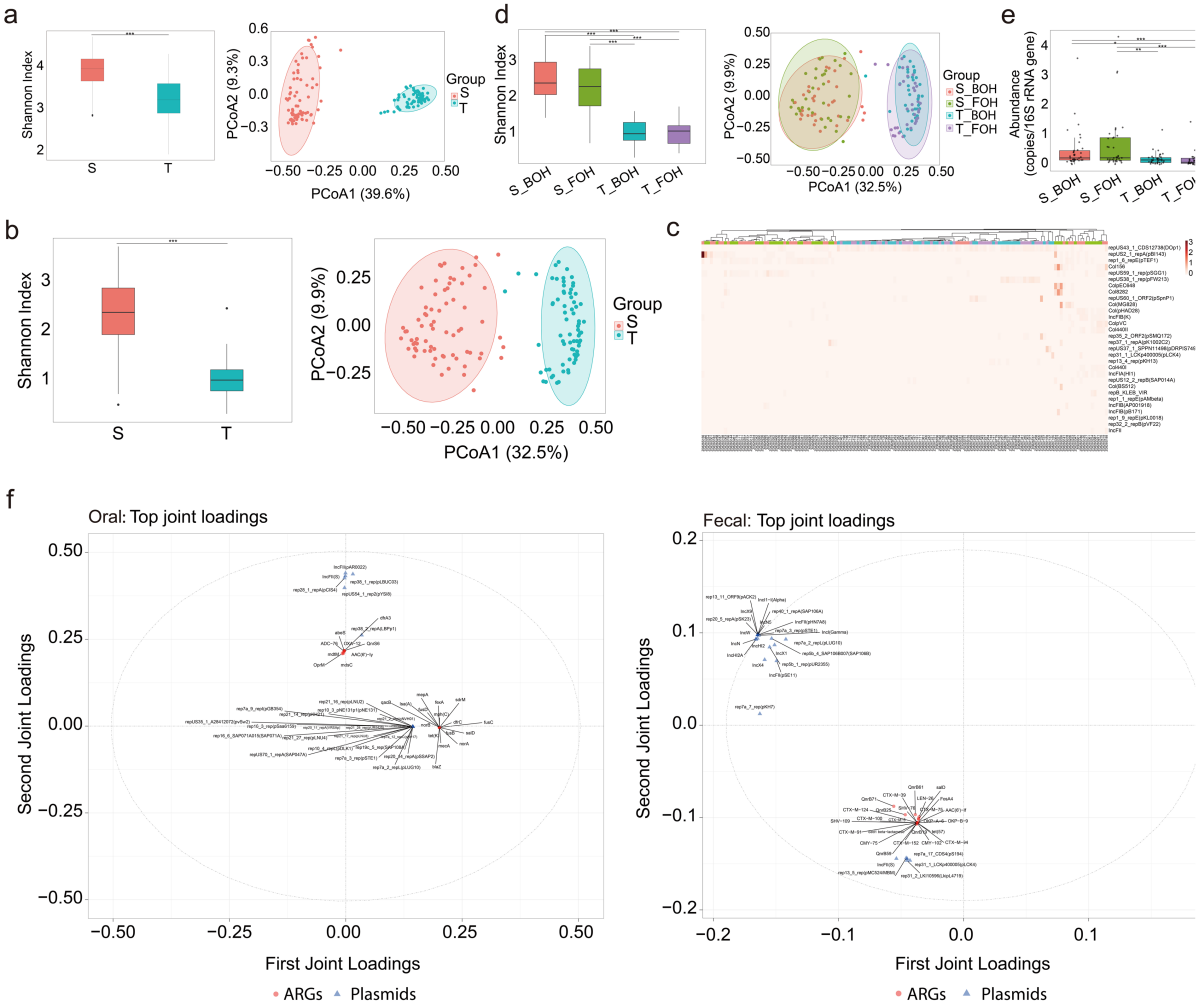
The distribution of mobile genetic elements (MGEs) and plasmids among the 144 samples (S: fecal, T: oral, S_BOH: fecal samples collected from back-of-house workers, S_FOH: fecal samples collected from front-of-house workers, T_BOH: oral samples collected from back-of-house workers, T_FOH: oral samples collected from front-of-house workers) (*0.01 < *p* < 0.05, **p < 0.01, ***p < 0.001). **(a)** The Shannon indices of MGEs (*χ*^2^ = 50.92, *p* < 0.001) and principal coordinate analysis (PCoA) of the MGEs (*R*^2^ = 0.38, *p* = 0.001) in the oral and fecal samples. **(b)** The Shannon indices of plasmids (*χ*^2^ = 83.65, *p* < 0.001) and PCoA of the plasmids (*R*^2^ = 0.29, *p* = 0.001) in the oral and fecal samples. **(c)** The relative abundance of the top 30 plasmids across all samples. **(d)** The Shannon indices of plasmids (S_BOH&S_FOH: *Z* = 0.90, *p_adj_* > 0.05; S_BOH&T_BOH: *Z* = 6.90, *p_adj_* < 0.001; S_BOH&T_FOH: *Z* = 7.01, *p_adj_* < 0.001; S_FOH&T_BOH: *Z* = 5.90, *p_adj_* < 0.001; S_FOH&T_FOH: *Z* = 6.02, *p_adj_* < 0.001; T_BOH&T_FOH: *Z* = 0.20, *p_adj_* > 0.05) and PCoA of the plasmids (S_BOH&S_FOH: *R*^2^ = 0.01, *p_adj_* = 0.403; S_BOH&T_BOH: *R*^2^ = 0.31, *p_adj_* = 0.001; S_BOH&T_FOH: *R*^2^ = 0.29, *p_adj_* = 0.001; S_FOH&T_BOH: *R*^2^ = 0.31, *p_adj_* = 0.001; S_FOH&T_FOH: *R*^2^ = 0.29, *p_adj_* = 0.001; T_BOH&T_FOH: *R*^2^ = 0.02, *p_adj_* = 0.158) in the four groups. **(e)** The plasmids abundance in the four groups. The abundance of plasmids was normalized to the abundance of the 16S rRNA gene and expressed as the number of plasmid copies per copy of the 16S rRNA gene (S_BOH&S_FOH: *Z* = −0.41, *p_adj_* = 0.68; S_BOH&T_BOH: *Z* = −2.55, *p_adj_* = 0.02; S_BOH&T_FOH: *Z* = −3.78, *p_adj_* = 0.0005; S_FOH&T_BOH: *Z* = −2.93, *p_adj_* = 0.007; S_FOH&T_FOH: *Z^2^* = −4.15, *p_adj_* = 0.0002; T_BOH&T_FOH: *Z* = −1.27, *p_adj_* = 0.24). **(f)** Loading plot of antimicrobial resistance genes (ARGs) subtypes and plasmids in the oral and fecal samples.

A total of 1,491 MGEs were identified. The diversity of MGEs in fecal samples was significantly higher than that in oral samples (*χ*^2^ = 50.923, *p* < 0.001). In addition, beta diversity analysis revealed distinct clustering patterns between oral and fecal samples (*R*^2^ = 0.38, *p* = 0.001) (Figure 5a). *Tn6216* was predominant in fecal samples, while *ISSpn14* dominated in oral samples (Supplementary Figure 7a). A total of 850 MGEs were overlapped between oral and fecal samples. Notably, 416 MGEs were exclusively detected in fecal samples, whereas 223 MGEs were unique to oral samples. In contrast, no significant differences were detected between different occupational groups from the same location (all *p_adj_* > 0.05) (Supplementary Figures 7b,c). The widespread prevalence of AMR is partly attributable to the horizontal transfer of ARGs, which is commonly mediated by plasmids within MGEs. A total of 335 plasmids were identified. Fecal samples exhibited significantly higher plasmid alpha diversity than oral samples (*χ*^2^ = 83.645, *p* < 0.001). Beta diversity analysis also revealed significant differences between the two sample types (*R*^2^ = 0.29, *p* = 0.001) (Figure 5b). In fecal samples, repUS2_1_repA(pBI143) was the predominant plasmid, while repUS43_1_CDS12738(DOp1) showed greater abundance in oral samples (Figure 5c). A total of 97 plasmids were overlapped between oral and fecal samples. Notably, 233 plasmids were exclusively detected in fecal samples, whereas only five plasmids were unique to oral samples. These results highlighted the intestinal tract as a highly active reservoir for plasmids. The total plasmid abundance, plasmid alpha and beta diversity did not differ significantly between occupational groups (all *p_adj_* > 0.05) (Figures 5d,e).

To further examine the relationships between ARGs, and BACs, MGEs, plasmids, the O2PLS method was applied. In both oral and fecal samples, plasmids showed a greater proportion of shared variation with ARGs than BACs or MGEs with ARGs (Supplementary Figures 8–11; Table 1), suggesting a relatively stronger association between plasmids and ARG profiles in both oral and gut environments (oral: *R*^2^_Y_ = 0.618; *R*^2^_Ycorr_ = 0.518; fecal: *R*^2^_Y_ = 0.408; *R*^2^_Ycorr_ = 0.291). The loading plots of the top 25 abundant plasmid and ARG subtypes further revealed that IncFII [oral: IncFII(pAR0022); fecal: IncFII(pHN7A8)] was the plasmid type most associated with ARG variation (Figure 5f).

**Table 1:** Contribution of bacterial communities (BACs), mobile genetic elements (MGEs), and plasmids to the orthogonal and associated components of antimicrobial resistance genes (ARGs) based on O2PLS analysis.

Class	Comparison type	R^2^_X_	R^2^_Y_	R^2^_Xcorr_	R^2^_Ycorr_
Oral	ARGs-BACs	0.306	0.386	0.198	0.21
	ARGs-MGEs	0.31	0.46	0.227	0.415
	ARGs-Plasmids	0.289	0.618	0.123	0.518
Fecal	ARGs-BACs	0.355	0.202	0.355	0.101
	ARGs-MGEs	0.399	0.355	0.399	0.273
	ARGs-Plasmids	0.451	0.408	0.346	0.291

## Discussion

In this study, we conducted a systematic comparative analysis of paired oral and fecal samples isolated from FOH and BOH workers. We provided an integrated characterization of the microbiome, pathogens, resistome, MGEs, and VFs across different human ecological niches, and further explored their potential links to food-service environment occupational exposure. Our study investigated the oral and gut microbiomes in Chinese restaurant workers, revealing significant differences between the oral and fecal microbiota. The gut microbiota exhibited higher taxonomic richness, diversity, and a higher pathogenic burden, whereas the oral microbiome was characterized by a smaller number of taxa with distinct occupation-associated features. At the phylum level, the dominant taxa in both oral and fecal samples were Firmicutes, Proteobacteria, Actinobacteria, and Bacteroidetes, consistent with previous studies (Ley et al., 2006). Prior research has reported that around 50% of species overlap between the oral cavity and feces in healthy individuals, whereas only 15.5% of gut microbes were shared with the oral microbiota in our samples, which may be attributable to the sampling site, as the alpha diversity of the buccal mucosa is lower than that of saliva or supragingival/subgingival plaque (Maki et al., 2021; Segata et al., 2012; Li et al., 2012). In healthy individuals, environmental and host factors preserve the site specificity of digestive tract microbiomes, permitting only a limited subset of oral microbes to colonize the gut (Schmidt et al., 2019). The increased relative abundance of oral bacteria in feces has two competing explanations: either oral bacteria invade the gut ecosystem and expand (the ‘expansion’ hypothesis), or oral bacteria transit through the gut and their relative increase marks the depletion of other gut bacteria (the ‘marker’ hypothesis) (Liao et al., 2024). Nonetheless, several strains were share/transfer between the oral and gut microbiota, which indicates that the oral and intestinal microbiota are not independent. Notably, among the genera overlapping between the oral and fecal samples, *Streptococcus*, *Veillonella*, and *Haemophilus* were also reported to be highly frequent transmitters in both sites (Schmidt et al., 2019). *S. salivarius* demonstrated notable probiotic potential, offering a promising strategy for preventing oral mucosal infections (Ding et al., 2025). Furthermore, clinical studies have linked the ectopic gut colonization of *V. parvula* to disease severity in patients with Crohn’s disease. Notably, this bacterium has been shown to actively exacerbate intestinal inflammation and promote *Clostridium difficile* infection (Yang et al., 2025). Periodontal disease is associated with increased gut colonization of pathogenic *H. parainfluenzae* in patients with Crohn’s disease. Intestinal colonization of the oral bacterium *H. parainfluenzae* has been associated with Crohn’s disease severity and progression (Sohn et al., 2023). These findings underscore the dual roles of strains overlapping between the oral and fecal samples in gastrointestinal health, acting as either protective agents or pathogenic drivers.

Antimicrobial-resistant ESKAPE pathogens posed a grave and escalating global threat to human health (De Oliveira et al., 2020). The acquisition and dissemination of ARGs among these pathogens have severely limited available treatment options for serious infections, amplified the burden of disease, and contributed to elevated mortality rates due to frequent therapeutic failure. In this study, members of the ESKAPE strains were identified in both oral and fecal samples. Although our data did not permit direct assessment of antimicrobial resistance phenotypes of isolates, the detection of ESKAPE taxa remains notable, as these pathogens are well recognized for their clinical relevance, high virulence, and frequent association with multidrug-resistant infections (Miller and Arias, 2024). Compared to the oral, the intestine carries a wider ESKAPE pathogens, making the gut more important for containing, even spreading ESKAPE pathogens. Previous evidence has demonstrated that the intestinal tract provides a favorable environment for horizontal gene transfer and the persistence of multidrug-resistant bacteria (Kent et al., 2020). Consequently, the gut microbiome should be the primary focus in surveillance and control strategies for ESKAPE pathogen transmission. In addition, occupational exposures can significantly shape the food processing workers and may impact their health status (Zegeye et al., 2025). Our study found that restaurant occupational exposure exerted a more pronounced effect on the oral microbiome, while the gut microbiome appears comparatively stable, likely due to its greater ecological stability (Chen et al., 2021). Notably, the beta diversity of VFs within the oral microbiota differed between the two occupational groups, indicating that occupational exposure may affect the VF of oral microbes. These oral VFs could serve as biomarkers to assess infection risk associated with different occupational environments, providing a direct scientific basis for targeted public health interventions. The exclusive detection of food-associated species (*L. carnosus*, *W. viridescens*) in BOH workers (S_BOH and T_BOH) further supported the influence of occupational environments on microbial composition. *W. viridescens*, an opportunistic pathogen, can cause systemic infections like bacteremia and endocarditis, with its frequent misidentification often leading to diagnostic delays and inappropriate treatment (Jamwal et al., 2024), suggesting that the risk of infection caused by occupational exposure to BOH workers should be given attention. The oral microbiota exhibited a particular sensitivity to occupational exposure, likely attributable to routine contact of BOH workers with raw food and kitchen environments. This positions the oral cavity as an overlooked potential reservoir and transmission route for pathogens, highlighting a novel target for public health intervention. Furthermore, the important foodborne pathogen *S.* Typhimurium (Majowicz et al., 2010) was identified in our study. These *S.* Typhimurium isolates may transiently exist in the oral cavity, a phenomenon observed more frequently among BOH workers, which is consistent with previous reports of higher *Salmonella* carriage in food workers (Lu et al., 2024). The transient presence of *S.* Typhimurium in the oral cavity and its occupational association indicates that BOH workers constitute a critical node in the foodborne transmission chain, consistent with the fecal-oral transmission route characteristic of this foodborne pathogen. During processes such as food washing, chopping, or high-heat cooking, bioaerosols containing foodborne pathogens can be generated. Therefore, the presence of *S.* Typhimurium in the oral cavity is likely due to short-term bioaerosol exposure. Although samples were collected during non-operational periods where food preparation was not occurring, these transient bioaerosols can remain suspended in the air and subsequently deposit on the oral mucosa, particularly if personal protective equipment (e.g., masks) was not strictly used. According to the regulations, BOH workers must wear masks when they deal with food processing, cooking, serving, delivery, takeaway services, ordering, cashiering. And we observed that staff generally complied strictly with these hygiene and personal protection requirements during working time. However, they do not consistently wear masks during periods when they are not handling food, although they remain in the workspace. We hypothesized that aerosols generated during food processing may persist in the environment, leading to a continuous exposure risk for BOH workers even during periods when they are not handling food. The gut carriage rate of *Salmonella* strains in healthy individuals has been reported to be low (1.91%) (Lu et al., 2024). Moreover, due to severe intestinal colonization bottlenecks, only a small fraction of ingested bacteria through the oral cavity can successfully colonize in the gut (Hotinger et al., 2025). These factors likely contribute to the non-detection of *S.* Typhimurium in the human gut despite occupational exposure. Therefore, proactive surveillance of pathogens in this BOH population should be prioritized, accompanied by targeted occupational health interventions, such as stringent enforcement of protective measures during food processing to effectively mitigate this potential transmission route.

The differences between oral and fecal samples, as well as the occupational variations, are not only reflected in microbial composition but also in the distribution of ARGs and MGEs. Compared to the oral microbiota, the gut microbiome not only harbors a higher diversity of ARGs and MGEs, particularly plasmids, but also carries more high-risk level ARGs. While both niches shared 30 Rank_I genes, the fecal samples uniquely harbored 23 additional Rank_I genes, including the broad-spectrum *β*-lactamase gene *bla*_CTX-M-55_, which is typically plasmid-mediated and can be transferred between different strains and hosts (Misumi et al., 2023; Kim et al., 2025; Li et al., 2022). In contrast, oral samples exclusively harbored only the *tet(G)* Rank_I gene. These findings hint at the gut as a key ecological niche for the accumulation of ARGs and horizontal gene transfer, where the high microbial density facilitates the exchange of MGEs and enhance the potential for resistance gene dissemination. Although there was no significant difference in overall ARG abundance between different occupational groups, the beta diversity of ARGs differed between the FOH and BOH workers in the oral samples. Some genes were only present in both the oral and fecal samples of the BOH workers, while others were shared only within the FOH workers, suggesting occupation-associated unique resistome features. Importantly, we identified a stable core of 30 high-risk Rank_I ARGs that were common to all groups across occupations and body sites. The widespread presence of these ARGs suggests strong ecological adaptability and potential horizontal gene transfer, indicating that they are less affected by ecological niches or occupational exposure and may therefore persist in the microbial communities of restaurant workers. Consequently, effective containment strategies should focus on environment section as key targets for interrupting ARG dissemination in one health framework. Furthermore, fecal samples exhibited higher alpha diversity of MGEs, and plasmids compared with oral samples, with significant differences in beta diversity between niches. This indicated that the gut microbiome not only harbors a broader spectrum of ARGs but also maintains a more complex network of MGEs, facilitating horizontal gene transfer and the dissemination of antimicrobial resistance (Broaders et al., 2013). O2PLS analysis revealed that plasmids exhibited the strongest associations with ARGs, compared to ARGs with BACs or MGEs, highlighting their potential role as key vectors in resistance dissemination. Plasmids are recognized as major vehicles of resistance dissemination, facilitating the transfer of ARGs across diverse bacterial hosts and thereby accelerating the spread of multidrug resistance across species and ecological boundaries (Wang and Dagan, 2024). These findings highlighted the central role of plasmids in ARG transmission and suggest that future containment strategies should evolve from traditional “bactericidal” approaches to precision “transmission-blocking” interventions (Buckner et al., 2018). Specifically, targeted elimination of plasmids carrying ARG represents a promising direction, and tailored intervention strategies may be required for different occupational groups within the community to effectively curb the spread of ARGs.

This study has several limitations. The sample size was relatively small and restricted to specific occupational groups, which may limit generalizability of the findings. Nevertheless, strain-level analysis was precluded for low-abundance taxa, thus limiting further strain-level validation. In restaurant environments, bacterial transmission can occur through multiple potential routes (including interactions between raw food and staff, staff and prepared food, customers and staff). However, as our sampling strategy did not incorporate a comprehensive ‘One Health’ (human-food-environment) framework, the precise transmission pathways remain to be fully elucidated. Additionally, unmeasured confounding factors may have influenced the observed pathobiomes and resistomes patterns. These limitations highlight the need for future studies with larger sample sizes and a full ‘One Health’ transmission chain design to more comprehensively investigate pathobiomes and resistomes transmission in restaurant.

In conclusion, this study demonstrates that the oral or gut microbiome of FOH and BOH workers in restaurant is significantly different due to distinct occupational exposure. Oral and gut microbiota/resistome features are shaped by occupational exposure in restaurants, and BOH workers play a crucial role in the transmission of pathogens and ARGs. Within FOH and BOH workers, the oral and gut microbiomes exhibit distinct ecological characteristics: the gut serves as a reservoir with higher microbial diversity, pathogenic burden, and accumulation of ARGs, MGEs, and VFs, whereas the oral cavity harbors fewer taxa but shows occupation-associated features. Despite these differences, the identification of clonal strains indicates that the oral and gut microbiomes are interconnected rather than independent. Analyses of ARGs indicate that plasmid-mediated horizontal gene transfer may contribute to shaping ARG profiles in both niches. Collectively, these findings underscore the need for targeted interventions, including targeted occupational health education and enhanced protective measures to limit pathogen transmission and curb the spread of AMR. These insights provide an evidence base for public health authorities and regulatory agencies to refine food safety protocols and occupational hygiene.

## Data Availability

The raw sequencing data have been deposited in the Sequence Read Archive (SRA) of the National Center for Biotechnology Information (NCBI) under BioProject accession number PRJNA1346870.

## Footnotes

1
https://github.com/biobakery/MetaPhlAn/wiki/Strain-Sharing-Inference

2
https://www.medrxiv.org/content/10.1101/2025.08.16.25333785v1
