# Supplementary material for: Restaurant occupational exposure affects the profiles of oral and gut pathobiomes and resistomes
Source: Front Microbiol. 2026 Feb 16;17:1771459. doi: 10.3389/fmicb.2026.1771459 (PMC12950533; doi:10.3389/fmicb.2026.1771459)
Supplement: Supplementary file 1 [file Data_Sheet_1.PDF]

Supplementary Figures

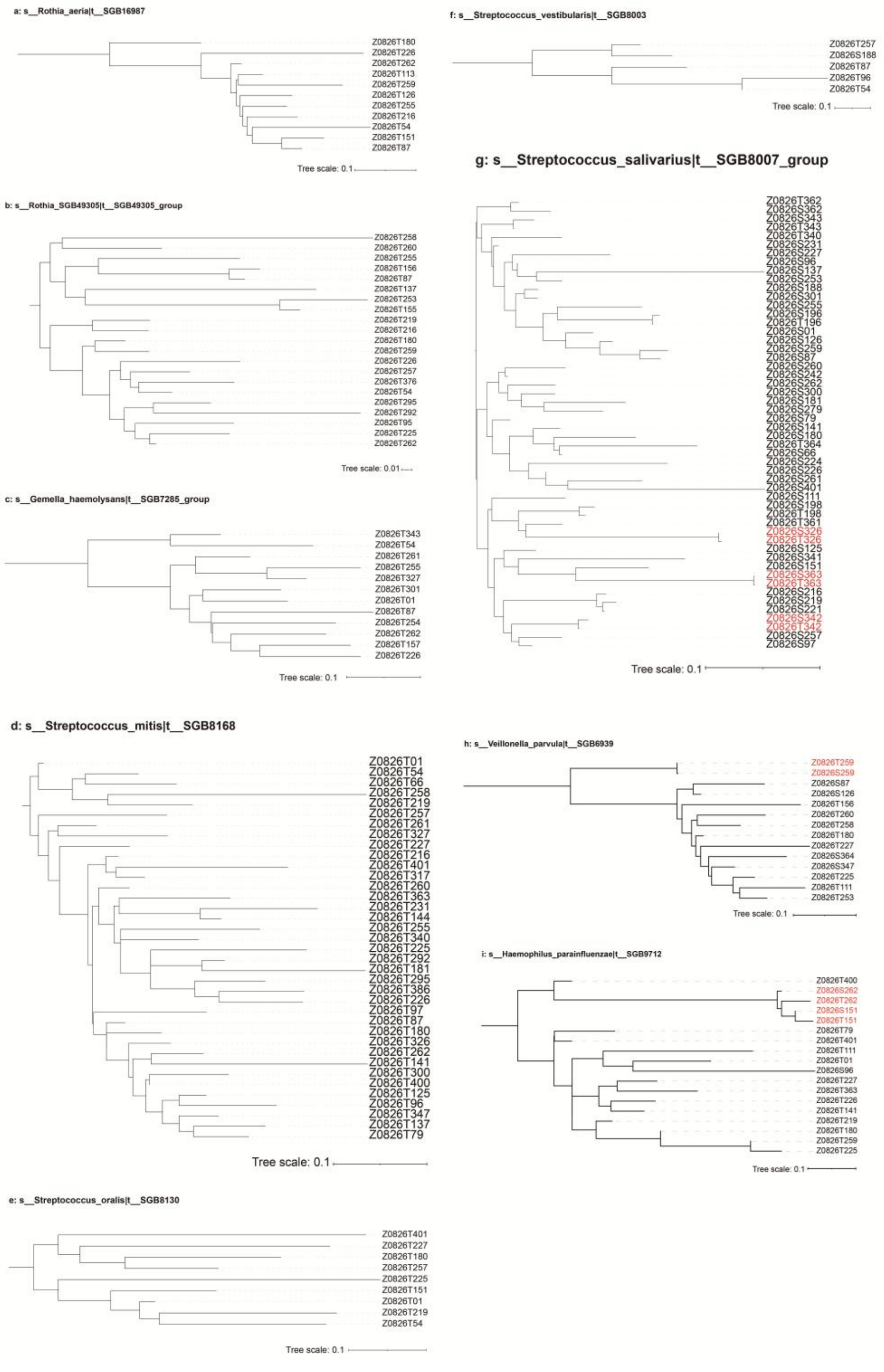

Supplementary Fig. 1 Phylogenetic trees produced by StrainPhlAn (S: fecal, T: oral). The red font indicates oral and fecal samples from the same individual with less than five SNP differences.

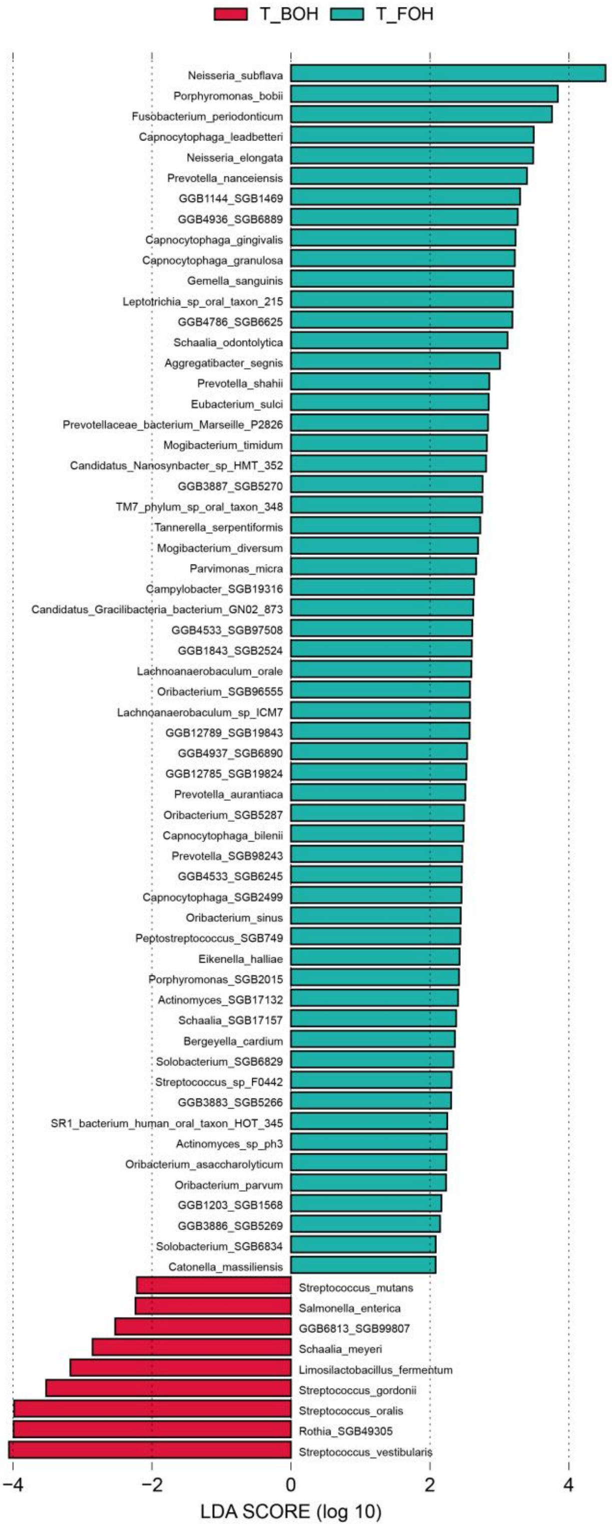

Supplementary Fig. 2 LEfSe analysis of species in oral samples from the T_BOH and T_FOH groups (T_BOH: oral samples collected from Back-of-House workers, T_FOH: oral samples collected from Front-of-House workers).

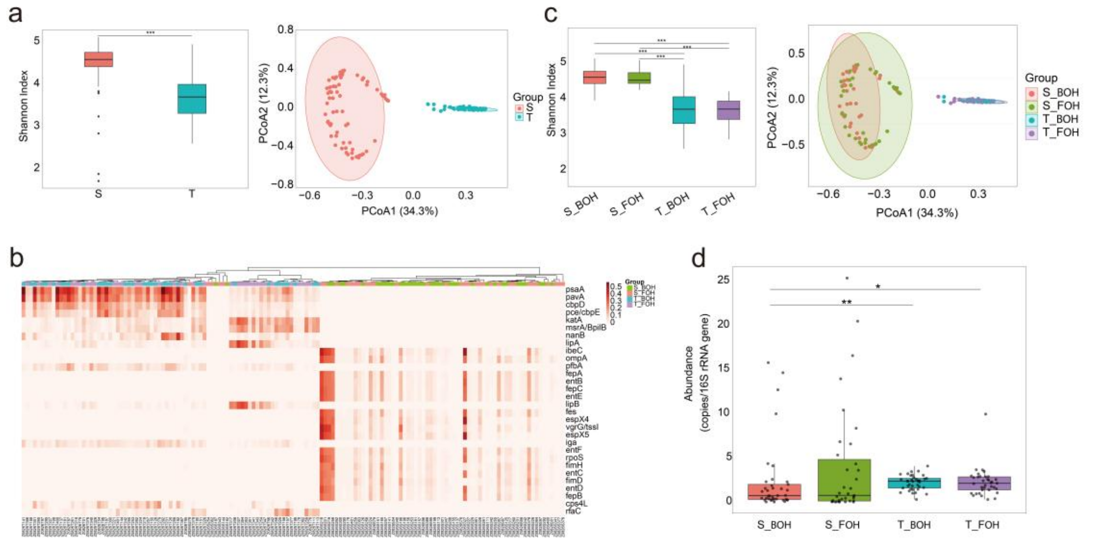

Supplementary Fig. 3 The distribution of virulence factors (VFs) among the 144 samples (S: fecal, T: oral, S_BOH: fecal samples collected from Back-of-House workers, S_FOH: fecal samples collected from Front-of-House workers, T_BOH: oral samples collected from Back-of-House workers, T_FOH: oral samples collected from Front-of-House workers) (*0.01 < p < 0.05, **p < 0.01, ***p < 0.001). **(a)** The Shannon indices of VFs (χ^2 = 74.621, p < 0.001) and principal coordinate analysis (PCoA) of the VFs (R^2 = 0.32, p = 0.001) in the oral and fecal samples. **(b)** The relative abundance of the top 30 VFs across all samples. **(c)** The Shannon indices of VFs (S_BOH&S_FOH: Z = 0.83, p_{adj} > 0.05; S_BOH&T_BOH: Z = 6.45, p_{adj} < 0.001; S_BOH&T_FOH: Z = 6.66, p_{adj} < 0.001; S_FOH&T_BOH: Z = 5.53, p_{adj} < 0.001; S_FOH&T_FOH: Z = 5.76, p_{adj} < 0.001; T_BOH&T_FOH: Z = 0.3, p_{adj} > 0.05) and PCoA of the VFs (S_BOH&S_FOH: R^2 = 0.02, p_{adj} = 0.08; S_BOH&T_BOH: R^2 = 0.37, p_{adj} = 0.001; S_BOH&T_FOH: R^2 = 0.35, p_{adj} = 0.001; S_FOH&T_BOH: R^2 = 0.32, p_{adj} = 0.001; S_FOH&T_FOH: R^2 = 0.30, p_{adj} = 0.001; T_BOH&T_FOH: R^2 = 0.03, p_{adj} = 0.019) in the four groups. **(d)** The VFs abundance in the four groups. The abundance of VFs was normalized to the abundance of the 16S rRNA gene and expressed as the number of VFs copies per copy of the 16S rRNA gene (S_BOH&S_FOH: Z = -0.76, p_{adj} = 0.89; S_BOH&T_BOH: Z = -3.07, p_{adj} = 0.01; S_BOH&T_FOH: Z = -2.62, p_{adj} = 0.04; S_FOH&T_BOH: Z = -2.26, p_{adj} = 0.09; S_FOH&T_FOH: Z = -1.83, p_{adj} = 0.20; T_BOH&T_FOH: Z = -0.407, p_{adj} = 0.89).

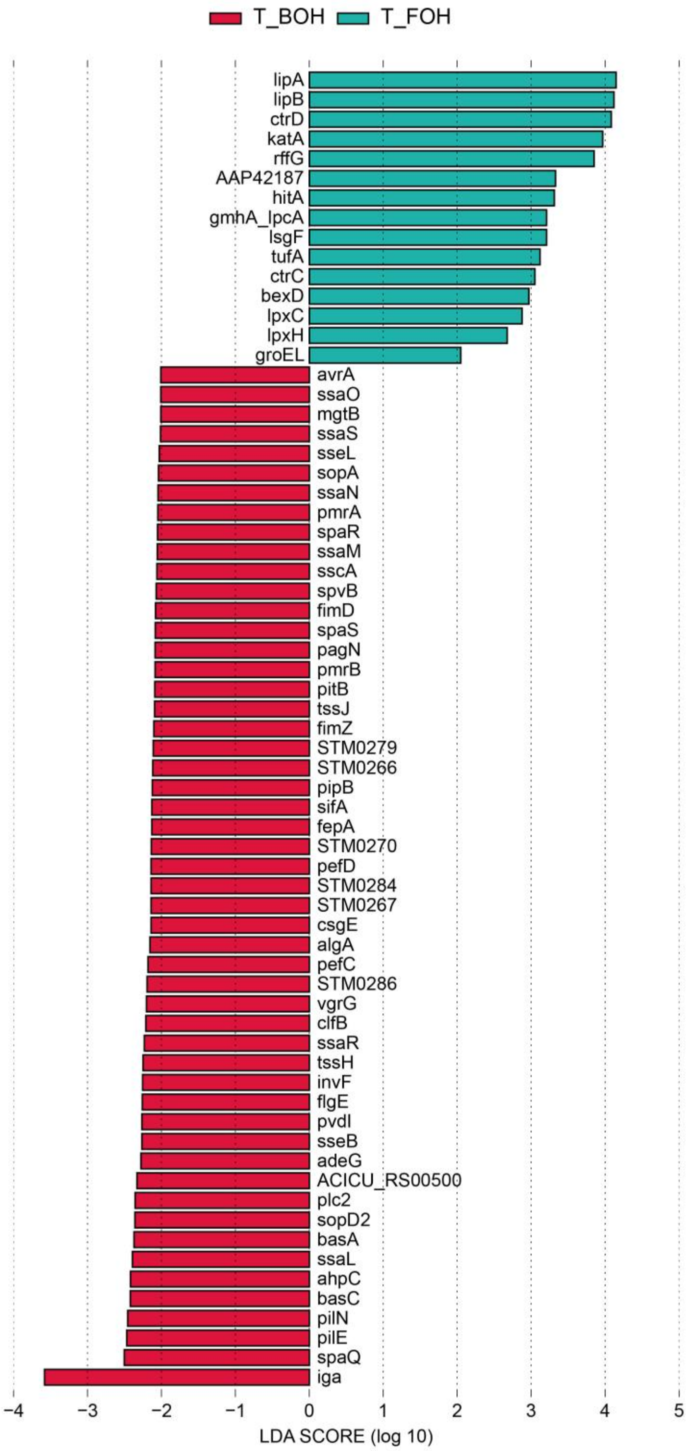

Supplementary Fig. 4 LEfSe analysis of VFs in oral samples from the T_BOH and T_FOH groups (T_BOH: oral samples collected from Back-of-House workers, T_FOH: oral samples collected from Front-of-House workers).

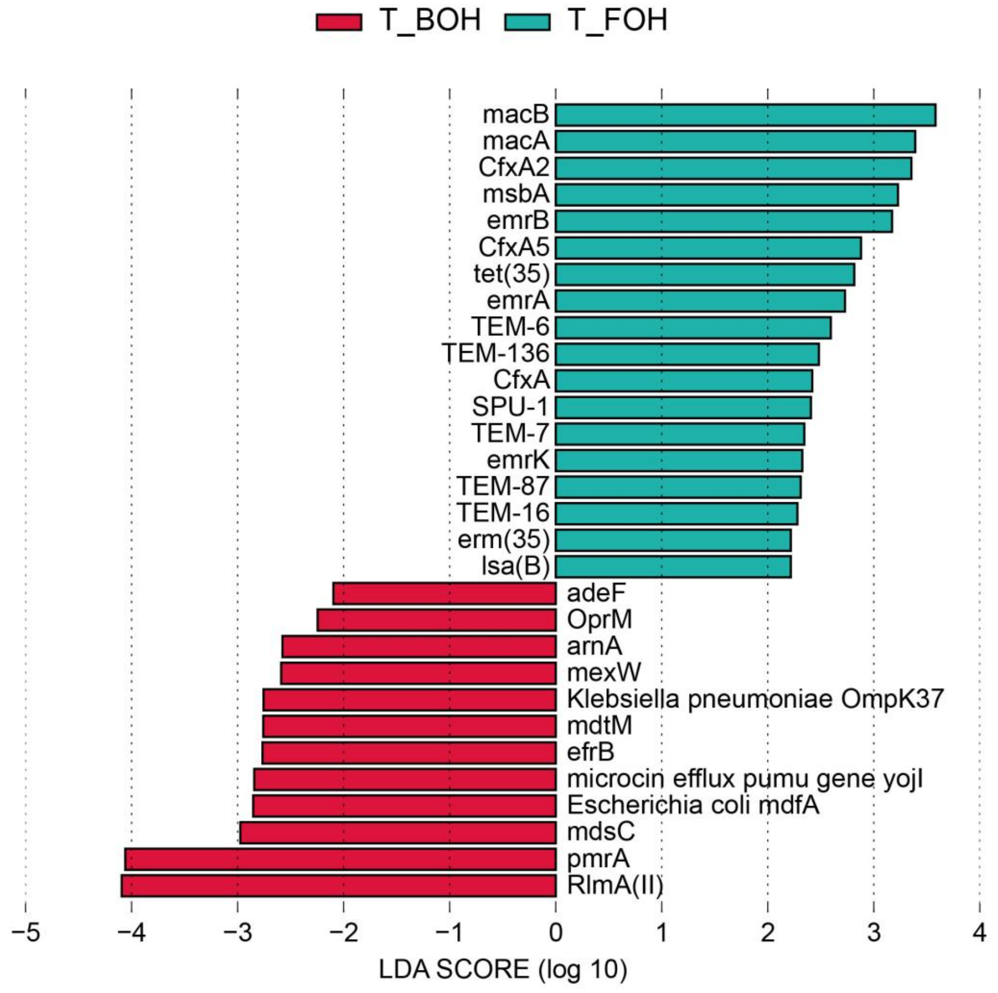

Supplementary Fig. 5 LEfSe analysis of ARGs in oral samples from the T_BOH and T_FOH groups (T_BOH: oral samples collected from Back-of-House workers, T_FOH: oral samples collected from Front-of-House workers).

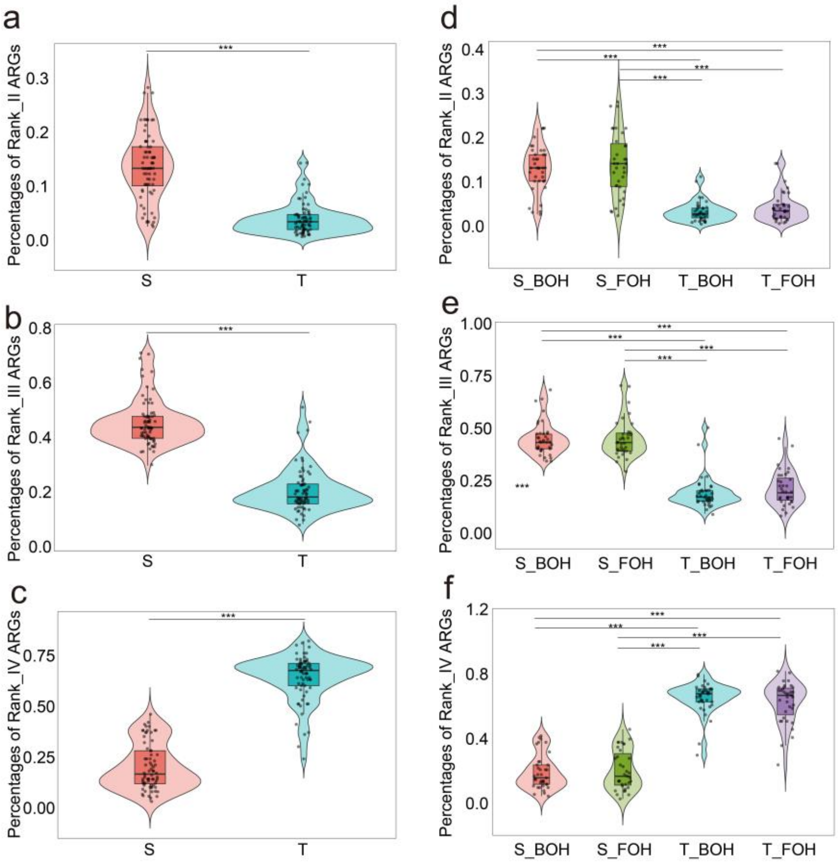

Supplementary Fig. 6 The percentages of Rank_II antimicrobial resistance genes (ARGs), Rank_III ARGs, and Rank_IV ARGs among the 144 samples (S: fecal, T: oral, S_BOH: fecal samples collected from Back-of-House workers, S_FOH: fecal samples collected from Front-of-House workers, T_BOH: oral samples collected from Back-of-House workers, T_FOH: oral samples collected from Front-of-House workers) (***$p < 0.001$). **(a)** The percentages of Rank_II ARGs among the oral and fecal samples ($w = 4759$, $p < 0.001$). **(b)** The percentages of Rank_III ARGs among the oral and fecal samples ($w = 66$, $p < 0.001$). **(c)** The distribution of Rank_IV ARGs among the oral and fecal samples ($w = 66$, $p < 0.001$). **(d)** The percentages of Rank_II ARGs in the four groups (S_BOH&S_FOH: $Z = -0.11$, $p_{adj} = 0.91$; S_BOH&T_BOH: $Z = 6.56$, $p_{adj} < 0.001$; S_BOH&T_FOH: $Z = 5.64$, $p_{adj} < 0.001$; S_FOH&T_BOH: $Z = 6.58$, $p_{adj} < 0.001$; S_FOH&T_FOH: $Z = 5.68$, $p_{adj} < 0.001$; T_BOH&T_FOH: $Z = -0.83$, $p_{adj} = 0.489$). **(e)** The percentages of Rank_III ARGs in the four groups (S_BOH&S_FOH: $Z = 0.12$, $p_{adj} = 0.90$; S_BOH&T_BOH: $Z = 7.34$, $p_{adj} < 0.001$; S_BOH&T_FOH: $Z = 6.60$, $p_{adj} < 0.001$; S_FOH&T_BOH: $Z = 7.12$, $p_{adj} < 0.001$; S_FOH&T_FOH: $Z = 6.39$, $p_{adj} < 0.001$; T_BOH&T_FOH: $Z = -0.64$, $p_{adj} = 0.624$). **(f)** The percentages of Rank_IV ARGs in the four groups (S_BOH&S_FOH: $Z = -0.10$, $p_{adj} = 0.924$; S_BOH&T_BOH: $Z = -7.45$, $p_{adj} < 0.001$; S_BOH&T_FOH: $Z = -7.01$, $p_{adj} < 0.001$; S_FOH&T_BOH: $Z = -7.25$, $p_{adj} < 0.001$; S_FOH&T_FOH: $Z = -6.82$, $p_{adj} < 0.001$; T_BOH&T_FOH: $Z = 0.34$, $p_{adj} = 0.884$).

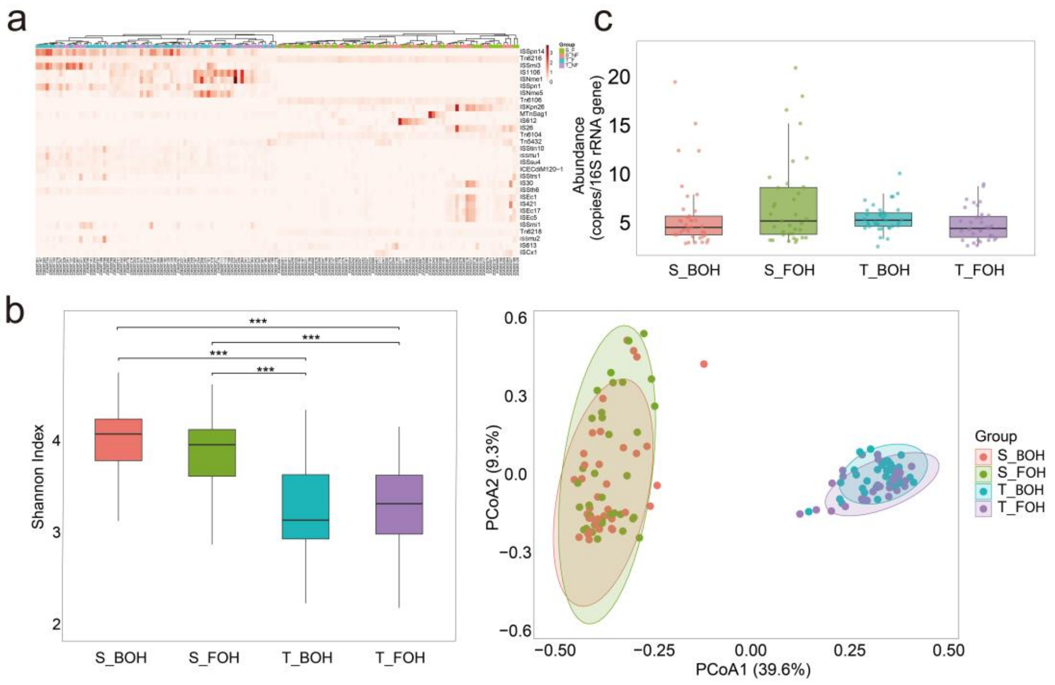

Supplementary Fig. 7 The distribution of mobile genetic elements (MGEs) among the 144 samples (S: fecal, T: oral, S_BOH: fecal samples collected from Back-of-House workers, S_FOH: fecal samples collected from Front-of-House workers, T_BOH: oral samples collected from Back-of-House workers, T_FOH: oral samples collected from Front-of-House workers) (***$p < 0.001$). **(a)** The relative abundance of the top 30 MGEs across all samples. **(b)** The Shannon indices of MGEs (S_BOH&S_FOH: $Z = 0.68$, $p_{adj} > 0.05$; S_BOH&T_BOH: $Z = 5.58$, $p_{adj} < 0.001$; S_BOH&T_FOH: $Z = 5.23$, $p_{adj} < 0.001$; S_FOH&T_BOH: $Z = 4.83$, $p_{adj} < 0.001$; S_FOH&T_FOH: $Z = 4.50$, $p_{adj} < 0.001$; T_BOH&T_FOH: $Z = -0.27$, $p_{adj} > 0.05$) and PCoA of the MGEs (S_BOH&S_FOH: $R^2 = 0.68$, $p_{adj} > 0.05$; S_BOH&T_BOH: $R^2 = 5.58$, $p_{adj} < 0.001$; S_BOH&T_FOH: $R^2 = 5.23$, $p_{adj} < 0.001$; S_FOH&T_BOH: $R^2 = 4.83$, $p_{adj} < 0.001$; S_FOH&T_FOH: $R^2 = 4.50$, $p_{adj} < 0.001$; T_BOH&T_FOH: $R^2 = -0.27$, $p_{adj} > 0.05$) in the four groups. **(c)** The MGEs abundance in the four groups. The abundance of MGEs was normalized to the abundance of the 16S rRNA gene and expressed as the number of MGEs copies per copy of the 16S rRNA gene (S_BOH&S_FOH: $Z = -1.41$, $p_{adj} = 0.47$; S_BOH&T_BOH: $Z = -1.65$, $p_{adj} = 0.39$; S_BOH&T_FOH: $Z = -0.58$, $p_{adj} > 0.05$; S_FOH&T_BOH: $Z = -0.22$, $p_{adj} > 0.05$; S_FOH&T_FOH: $Z = -1.97$, $p_{adj} = 0.25$; T_BOH&T_FOH: $Z = -2.21$, $p_{adj} = 0.16$).

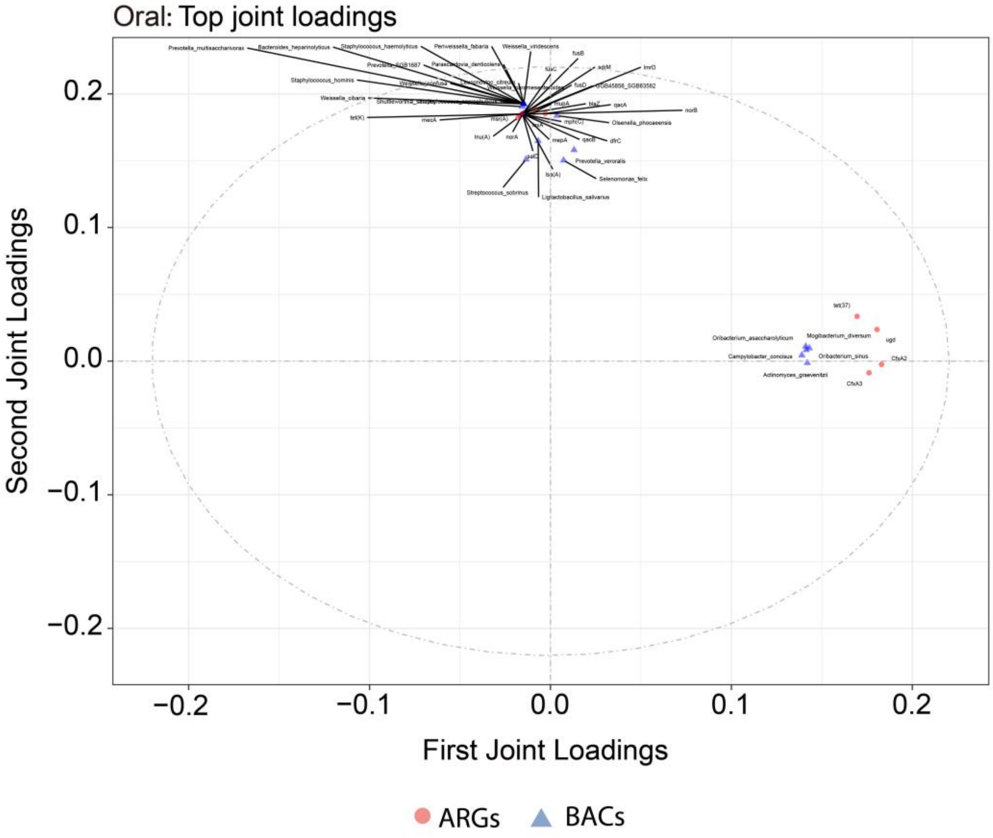

Supplementary Fig. 8 Loading plot of antimicrobial resistance genes (ARGs) subtypes and bacterial communities (BACs) in the oral samples.

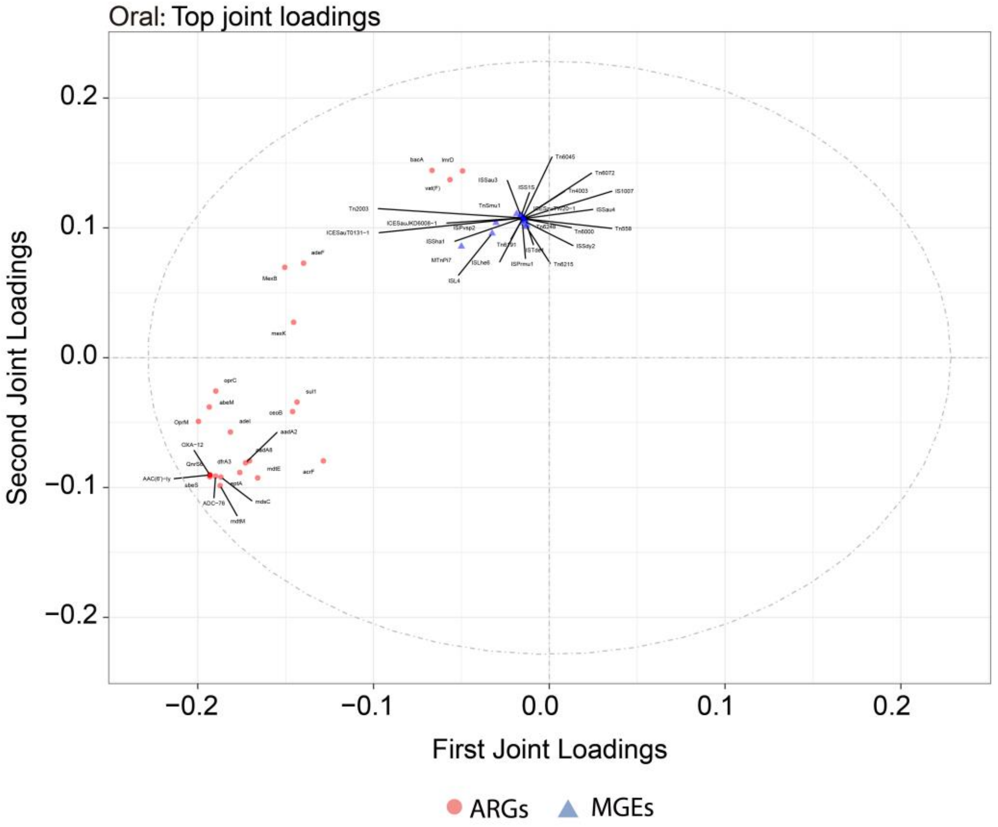

Supplementary Fig. 9 Loading plot of antimicrobial resistance genes (ARGs) subtypes and mobile genetic elements (MGEs) in the oral samples.

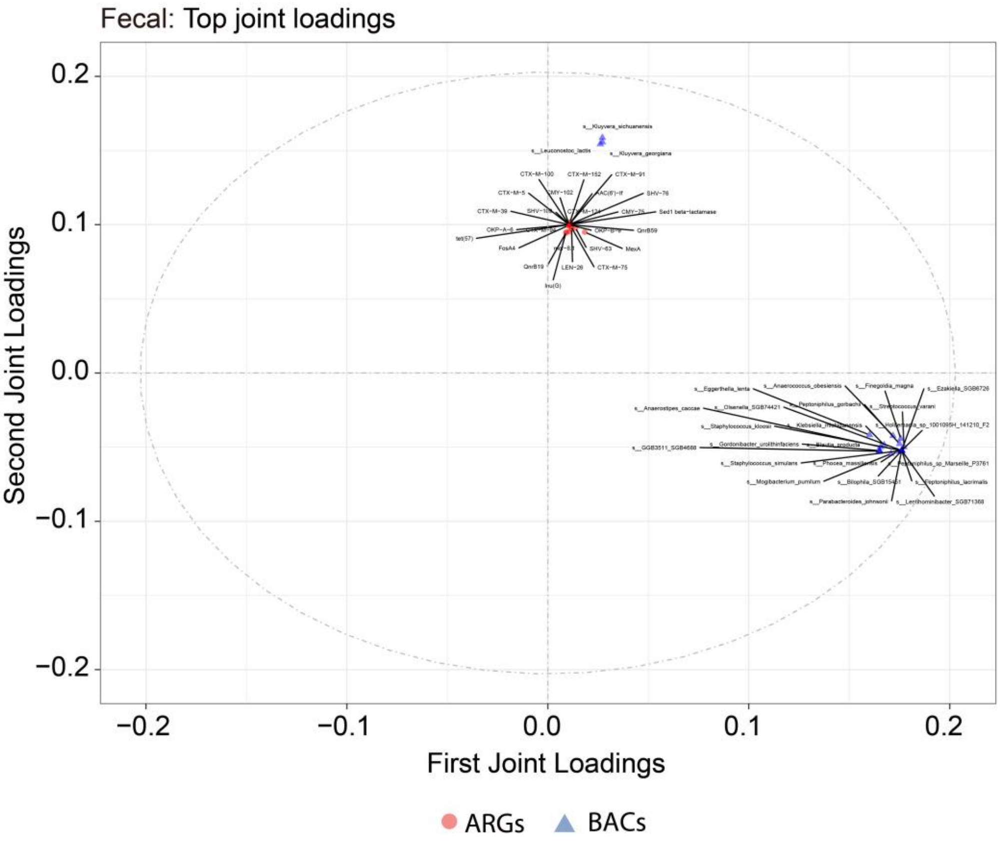

Supplementary Fig. 10 Loading plot of antimicrobial resistance genes (ARGs) subtypes and bacterial communities (BACs) in the fecal samples.

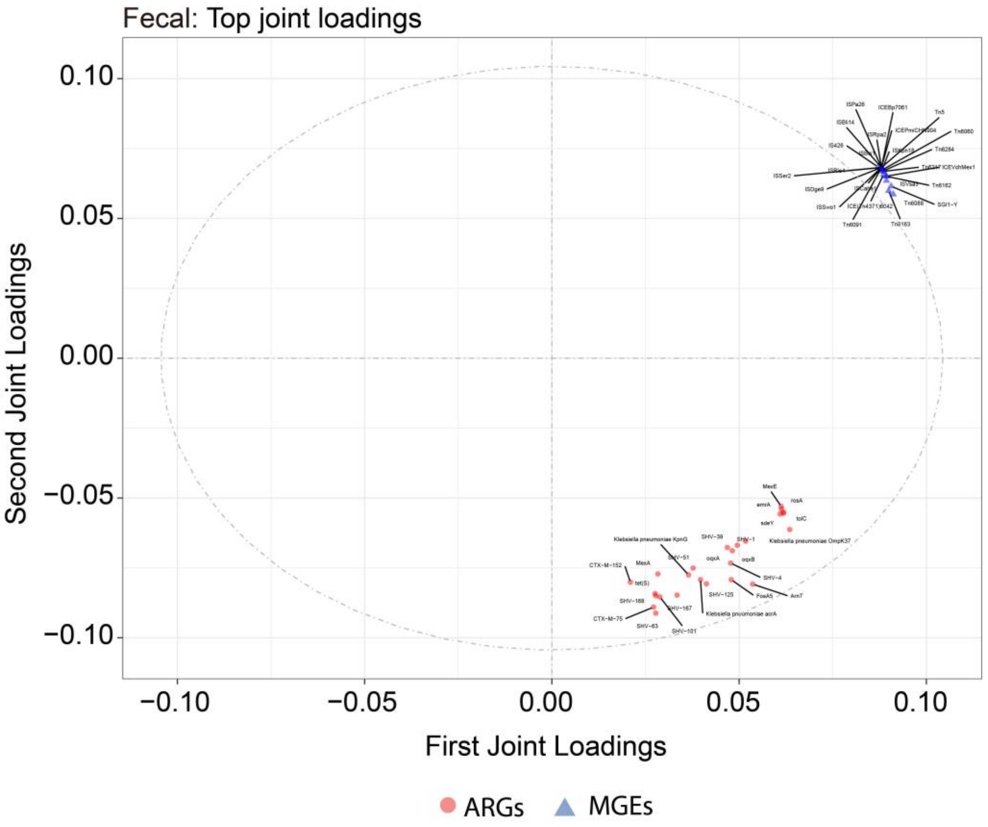

Supplementary Fig.11 Loading plot of antimicrobial resistance genes (ARGs) subtypes and mobile genetic elements (MGEs) in the fecal samples.
